# Use of a Small Animal Radiation Research Platform (SARRP) facilitates analysis of systemic versus targeted radiation effects in the mouse ovary

**DOI:** 10.1186/s13048-018-0442-8

**Published:** 2018-08-30

**Authors:** Allison R. Grover, Bruce F. Kimler, Francesca E. Duncan

**Affiliations:** 1 Center for Reproductive Science, Northwestern University, Chicago, IL 60611 USA; 2 Department of Radiation Oncology, Kansas University Medical Center, Kansas City, KS 66160 USA; 3 Department of Obstetrics and Gynecology, Feinberg School of Medicine, Northwestern University, 303 E. Superior Street, Lurie 7-117, Chicago, IL 60611 USA

**Keywords:** Ovary, Follicle, Radiation, Total body irradiation, Fertility preservation, latrogenic

## Abstract

**Background:**

Radiation exposure is known to cause accelerated aging and damage to the ovary, but the contribution of indirect versus direct effects is not well understood. We used the Small Animal Radiation Research Platform (SARRP) (Xstrahl) to deliver radiation to precise fields equivalent to clinical practice, allowing us to investigate systemic versus targeted damage in a structure as small as the mouse ovary. The X-ray dose was kept constant at 1 Gy, but the field varied. Mice either received total body irradiation (TBI), radiation targeted to both ovaries (T2), or radiation targeted to one ovary (left) while the contralateral ovary (right) was spared (T1). Sham mice, handled similarly to the other cohorts but not exposed to radiation, served as controls. Two weeks post-exposure, ovaries were harvested and analyzed histologically to identify and count follicles within each ovary.

**Results:**

Radiation significantly reduced primordial follicles in the TBI and T2 cohorts compared to the Sham cohort. There were no significant differences between these two irradiated groups. These findings suggest that at 1 Gy, the extent of damage to the ovary caused by radiation is similar despite the different delivery methods. When investigating the T1 cohort, targeted ovaries showed a significant decrease in primordial and growing follicles compared to non-targeted contralateral ovaries.

**Conclusions:**

These findings demonstrate that the SARRP is an effective strategy for delivering precise ionizing radiation to small organs such as mouse ovaries. Such tools will facilitate identifying the relative risks to ovarian function associated with different radiation fields as well as screening the efficacy of emerging fertoprotective agents.

**Electronic supplementary material:**

The online version of this article (10.1186/s13048-018-0442-8) contains supplementary material, which is available to authorized users.

## Background

Within the United States, more than 120,000 women under the age of 45 are diagnosed with cancer every year [1]. The three-pronged approach of surgical resection, chemotherapy, and radiation therapy has been a mainstay of cancer treatment [2, 3]. Although these approaches are lifesaving, they can have unintended off target health consequences. For example, exposure to radiation can accelerate reproductive aging and entry into menopause [4, 5]. This accelerated reproductive aging in turn can lead to subfertility, infertility, or increased complications during pregnancies that are achieved [6, 7]. As cancer treatments improve, survivorship is projected to increase to 20.3 million people in the United States by 2026, representing a 4 million increase in survivors in the span of a decade [2, 3, 8]. Thus, off target effects of treatment that may have detrimental consequences on these survivors’ overall health and quality of life must be addressed.

Radiation therapy is a delicate balance between destruction of malignant cells and minimizing damage to healthy tissue, but it can unintentionally damage key organs of the reproductive system. For example, in treatment of acute myeloid leukemia and conditioning therapy for bone marrow transplantation in Hodgkins and non-Hodgkins lymphoma, total body irradiation exposes the whole body to radiation, including the hypothalamus-pituitary-gonad axis (HPG axis) [2, 3, 9]. When the HPG axis is targeted, hormone secretion such as gonadotropin pulses can be perturbed leading to amenorrhea, hypogonadism, precocious puberty, and other endocrine disruptions [10–13]. The uterus may be exposed to radiation in cases where there is a uterine tumor or a solid abdominal tumor, and in both scenarios, the uterus would be in the radiation field [5, 14]. Radiation exposure can result in uterine vascular insufficiency and decreased uterine volume [9, 11, 14]. When the ovary is exposed to radiation through total body or abdominal radiation, oocytes are lost through cell death pathways; granulosa cell damage results in impaired gonadal hormone production; and the ovarian vasculature and stroma are compromised. These changes may ultimately lead to premature ovarian failure, subfertility, or infertility [5, 6, 9, 11, 12, 14].

From a mechanistic perspective, radiation primarily affects cells via direct induction of DNA damage, including base damage, base losses, single-strand breaks, double-strand breaks, and DNA-protein crosslinks [15, 16]. Such damage leads to activation of DNA damage repair pathways that if not repaired properly can result in cell death, cell transformation, or heritable genetic mutations [15, 17]. Irradiated cells may also initiate damage signals that affect nearby non-irradiated cells, and this phenomenon is referred to as the radiation-induced bystander effect (RIBE) [18, 19]. The RIBE then induces several cellular responses in the non-irradiated cells including oxidative DNA damage and inflammatory networks [20, 21]. Importantly, the RIBE appears to dominate at low dose radiation exposures (< 1 Gy) and thus may be a significant contributor to ovarian damage [22, 23]. Finally, there are abscopal effects where radiation induces damage to non-irradiated tissues and organs that are remote from the site of exposure [15]. For example, radiation restricted to the hypothalamus or pituitary may result in effects observable in the ovaries [9].

Of all reproductive organs, the ovary – including both its somatic and germ cell compartments – is particularly susceptible to radiation damage. In fact, the radiation dose required for 50% loss of function (LD_50_ value) in the human oocyte is estimated at < 2 Gy, but oocyte radiosensitivity is also highly dependent on species, developmental stage, and animal age [6, 24, 25]. In some mouse strains, the LD_50_ value has been reported to be as low as 0.15 Gy, and in both mouse and rat, oocytes within primordial follicles appear to be much more radiosensitive relative to the larger follicle stages [6, 26]. The DNA damage checkpoints and cell death pathways that are elicited in response to radiation exposure have been extensively investigated in the oocyte [6, 27]. However, whether or not radiation damage in the ovary is due solely to direct and targeted effects or to systemic effects has been difficult to distinguish, especially in animal model systems such as the rodent, because of the technical limitations in the ability to administer precise radiation doses to small fields [28–30]. The mouse ovary represents a significant challenge for targeted irradiation methods because, compared to the human ovary, it is substantially smaller and has a different anatomy [31]. The mouse ovary is surrounded by a bursa, a layer of epithelial cells that isolates the ovary from the abdominal cavity, it is located caudal to the kidneys instead of in the pelvic cavity, and it is connected to a uterine horn via the oviduct [31–33].

Recently, innovative microirradiators that mimic the treatment conditions used in modern clinical radiation oncology facilities and veterinary practices have been developed for use in small animals [34, 35]. These platforms can achieve targeting and imaging accuracy to 0.1 mm resolution, thus enabling radiation-based preclinical and basic science studies [35, 36]. In this study, we used the Xstrahl Small Animal Radiation Research Platform (SARRP) to compare ovarian damage due to a single low dose of ionizing radiation in three cohorts of reproductively adult mice: one that received total body irradiation (TBI), one that received targeted radiation to both ovaries, and one that received targeted radiation to one ovary while sparing the contralateral ovary. We found that both low dose TBI and targeted radiation caused a similar significant reduction in follicle numbers and that off target effects were negligible. These findings provide proof-of-concept that the SARRP is an effective means of delivering precise radiation to organs as small as a mouse ovary and an innovative tool to explore and decipher mechanisms of targeted versus systemic radiation-induced damage to reproductive organs.

## Results

### Use of the SAARP to examine systemic versus targeted effects of ionizing radiation on ovarian tissue

**Fig. 1:**
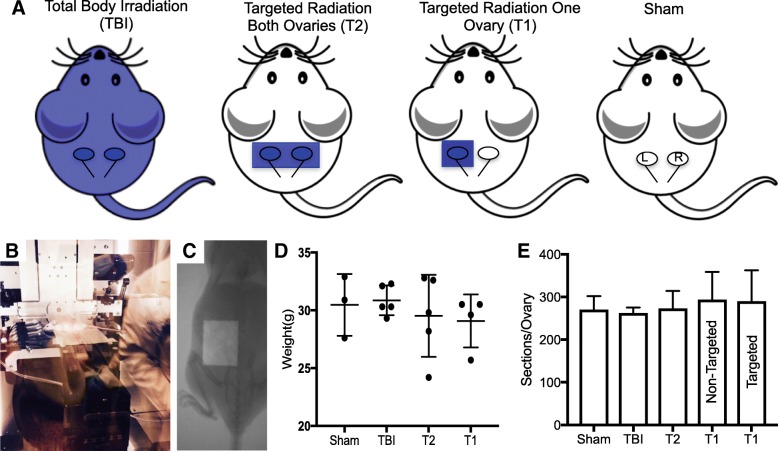
**Use of the SAARP to examine the effect of differences in radiation field on ovarian damage**. **a** A schematic of the experimental paradigm. Adult female mice received a single X-ray dose of 1 Gy and ovaries were harvested 2 weeks post-exposure. The Total Body Irradiation (TBI) cohort received the dose to the entire organism (*n* = 5). The targeted radiation cohort to both ovaries (T2) received the radiation dose to a 20 mm × 10 mm field encompassing both ovaries (*n* = 5). The targeted radiation cohort to one ovary (T1) received the radiation to a 10 mm × 10 mm field focused on the left ovary (*n* = 4). The Sham cohort underwent the same procedures as the experimental cohorts but was not irradiated (*n* = 3). **b** A representative image of how a mouse was anesthetized for immobilization in the beam field of the SARRP to target the ovaries. **c** A representative image that highlights the specified area of the collimating X-ray beam that was used to target the field of a single ovary. **d** Mouse weights were monitored 2 weeks post-exposure prior to euthanasia to examine whether there were acute health effects due to radiation. A one-way ANOVA test was performed and no differences were observed in weight between the groups (*p* = 0.73). **e** The total number of 5 μm serial sections generated per ovary was averaged and used as a proxy for ovarian size. A one-way ANOVA test was performed and no differences were observed between the groups (*p* = 0.68)

We have previously demonstrated that a single dose of 1 Gy TBI is sufficient to cause a significant reduction in ovarian follicles 2 weeks post-exposure in adult female CD1 mice, whereas a lower dose of 0.1 Gy is not [37]. Consequently, we exposed mice to a single dose of 1 Gy radiation but varied the field (Fig. 1a-c). We used the SAARP to generate three experimental cohorts of mice: one that received TBI, one that received targeted ionizing radiation to both ovaries, and one that received targeted radiation to a single ovary while the contralateral ovary was spared (Fig. 1a). Comparison of ovaries from mice that received TBI to those that were exposed to a targeted radiation field containing both ovaries allowed us to investigate systemic damage relative to targeted damage. In addition, by comparing targeted ovaries to contralateral non-targeted ovaries within individual animals, we were able to assess off-target effects in a paired organ system. For all cohorts, ovaries were harvested 2 weeks post-exposure. Importantly, we did not observe significant differences in animal weight among the cohorts at the time of tissue harvest (Fig. 1d). This consistency in weight in addition to the absence of animal lethality, suggests that the exposures to 1 Gy did not cause acute toxicity at this time point. We also did not observe any difference in the total number of serial sections obtained per ovary, suggesting that radiation exposure did not cause dramatic changes in ovarian size (Fig. 1e).

### Comparison of ovarian damage following total body irradiation versus targeted radiation to both ovaries

**Fig. 2:**
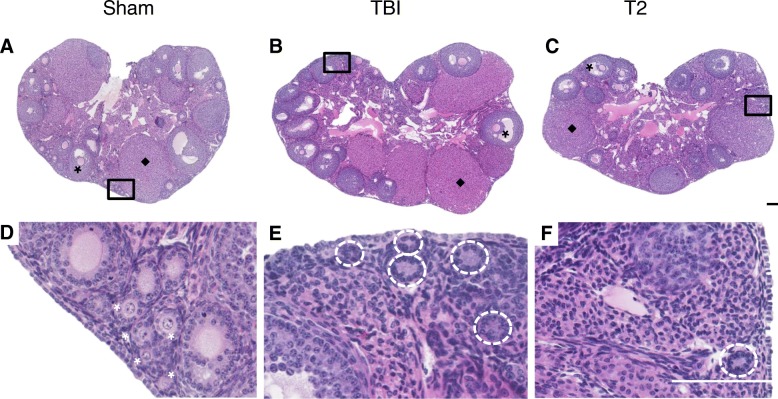
**TBI and targeted radiation to the ovary with X-rays at a dose of 1 Gy cause ovarian damage that is evident by histology.** Representative images of whole histological sections of ovaries from the following cohorts are shown: (**a**) Sham, (**b**) TBI, and (**c**) targeted radiation to both ovaries (T2). Examples of healthy antral follicles are highlighted with black asterisks and corpora lutea are highlighted with black diamonds. Corresponding higher magnification images of the boxed regions are shown in (**d-f**) to visualize primordial and primary follicles at the ovarian cortex. White asterisks in (**d**) highlight specific primordial and primary follicles that are absent in (**e**) and (**f**). Remnants of primordial and primary follicles that lack oocytes are shown with dashed circles in (**e**) and (**f**) in the TBI and T2 cohorts. The scale bars are 125 μm

**Fig. 3:**
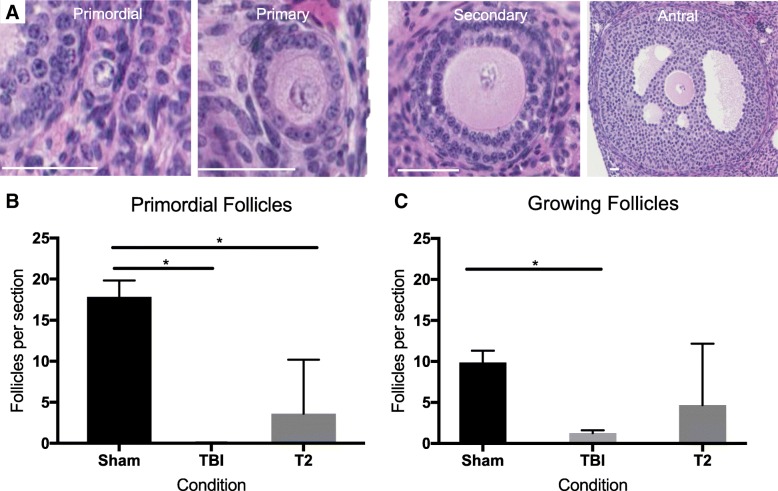
**TBI and targeted radiation to the ovary with X-rays at a dose of 1 Gy cause significant follicle loss. a** Follicle classes were defined according to morphological criteria. Primordial follicles were identified as an oocyte surrounded by squamous granulosa cells. Primary follicles were defined as an oocyte surrounded by one layer of cuboidal granulosa cells. Secondary follicles were defined as an oocyte surrounded by two or more layers of cuboidal granulosa cells. Antral follicles were defined as an oocyte surrounded by multiple layers of cuboidal cells with an antral space filled with follicular fluid forming in the middle of the follicle. Growing follicles were considered to be follicles at the primary stage or beyond. The average number of (**b**) primordial follicles per section and (**c**) growing follicles per section were quantified in ovaries from each cohort (Sham, TBI, and T2). A one-way ANOVA was performed between cohorts, and statistical significance was defined as *p < 0.05. The scale bar is 50 μm

To examine the ovarian effects of systemic versus targeted radiation, we compared the ovaries from the animals that received TBI and targeted radiation to both ovaries (T2) to each other and to the Sham controls (Fig. 1). Ovarian histological sections were used to evaluate tissue appearance and to perform quantitative analysis of healthy follicles. Analysis of histological sections revealed that ovaries from all cohorts, irrespective of radiation exposure, had morphologically normal antral follicles and corpora lutea providing evidence of both follicle growth and estrous cyclicity (Fig. 2a-c). Whereas primordial and primary follicles were clearly visible in ovarian sections from the Sham cohort, they were not visible in the irradiated cohorts (Fig. 2d-f). In fact, in ovarian sections from both the TBI and T2 cohorts, only remnants of small follicles were visible, and these were not included in the follicle counts (Fig. 2e and f). To quantify these differences between the experimental groups and the Sham control, we classified and counted healthy follicles according to previously established morphological criteria (Fig. 3a) [38]. When examining all follicle classes together, both TBI and T2 cohorts showed a significant decrease in total counts relative to the Sham cohort (343.5 ± 92.0 follicles, 1761.0 ± 2620.3 follicles, and 6689.2 ± 2289.6 follicles, respectively; Additional file 1). We were particularly interested in primordial follicles, since they dictate the ovarian reserve and reproductive lifespan of an individual. Thus, we examined the number of primordial follicles in each experimental cohort separately from growing follicles. Within the Sham cohort, there was an average of 17.8 ± 2.0 primordial follicles per section (Fig. 3b) and 9.9 ± 1.4 growing follicles per section (Fig. 3c). Exposure to radiation significantly reduced the amount of average primordial follicles to 0.1 ± 0.1 follicles per section in the TBI cohort and 3.6 ± 6.6 follicles per section in the T2 cohort (Fig. 3b). Exposure to radiation also reduced the number of growing follicles to an average of 1.3 ± 0.3 growing follicles per section in the TBI cohort and an average of 4.7 ± 7.5 follicles per section in the T2 cohort (Fig. 3c). However, this reduction was only significant when comparing the TBI to the Sham cohort (Fig. 3c). If we further examined growing follicles on a class-specific basis, we observed that there were significantly fewer primary and secondary follicles in both the TBI and T2 cohorts relative to the Sham controls, but there were no differences in the number of antral follicles across cohorts, suggesting that the largest follicles are not sensitive to radiation-induced damage (Additional file 2).

**Fig. 4:**
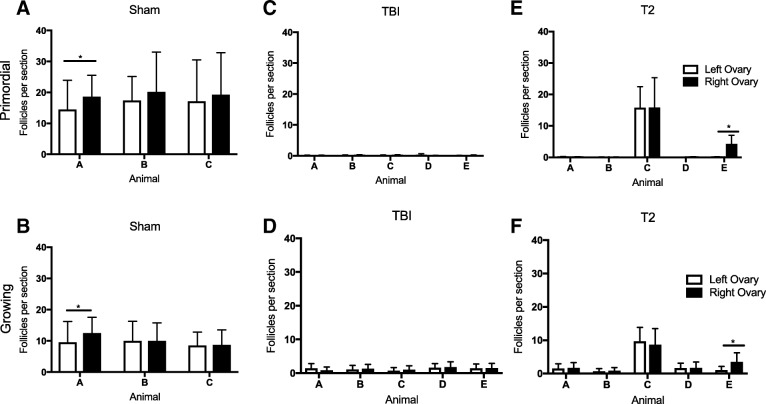
**Individual animal variation in ovarian follicle numbers in response to radiation damage.** The follicle data shown in Fig. 3 was further broken down by individual animal (letter) and by right (black) and left (white) ovaries for the (**a-b**) Sham (**c-d**) TBI, and (**e-f**) T2 cohorts. Data for primordial follicles are shown in (**a**, **c**, **e**) and data for growing follicles are shown in (**b**, **d**, **f**). Unpaired t-tests were performed with statistical significance defined as *p < 0.05

Because we kept the ovaries from each individual animal separate for processing, we were able to further analyze the data to see if there were any differences in follicle counts depending on the animal or the respective ovary (Fig. 4, Additional file 3). In the Sham cohort, both primordial and growing follicle counts were similar across mice (Fig. 4a and b). The follicle counts between left and right ovaries were similar in two of the three mice, but in one mouse, the right ovary had significantly more follicles per section than the left (18.6 ± 6.9 and 14.5 ± 9.4 primordial follicles per section, respectively, and 12.5 ± 5.0 and 9.6 ± 6.7 growing follicles per section, respectively). In the TBI cohort, follicle counts were similarly low across all mice, and no differences were seen between the left and right ovaries in both primordial and growing follicle counts (Fig. 4c and d). In the T2 cohort, three of the five mice exhibited a near loss of all primordial and growing follicles in response to radiation, but two mice still had follicles present (Fig. 4e and f). In fact, one of the animals (mouse C) had follicle counts that were similar to those in the Sham cohort (Fig. 4a and c). Thus, the observation that mice in the T2 cohort had a less pronounced reduction in follicle numbers compared to the TBI cohort was primarily due to the response of two animals rather than a global phenomenon across the cohort (Fig. 3b-c and Fig. 4c-f). Specifically, mouse C and E still had primordial follicles present (Fig. 4e). Also in mice from the T2 cohort, all the right and left ovaries had similar numbers of follicles except for one mouse in which the right ovary had more follicles than the left (4.3 ± 2. and 0.0 ± 0.3 primordial follicles per section, respectively; Fig. 4e and 3.5 ± 2.7 and 1.1 ± 1.1 growing follicles per section, respectively, Fig. 4f).

### Comparison of ovarian damage following targeted radiation to a single ovary relative to the non-targeted contralateral ovary

**Fig. 5:**
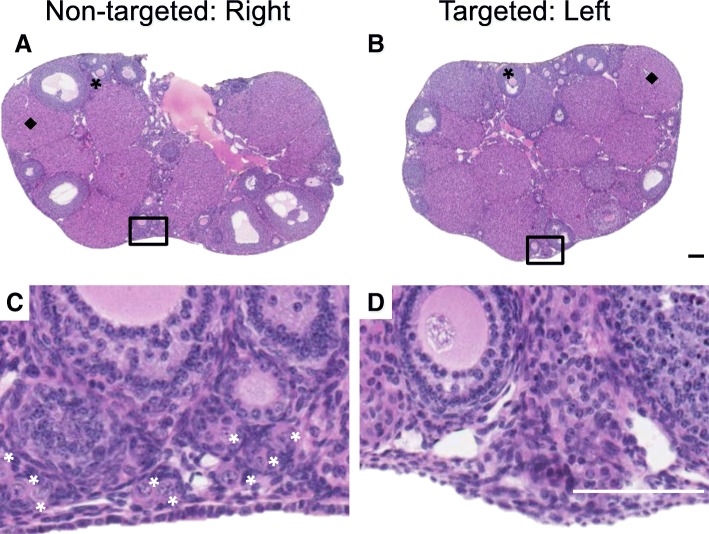
**Histological evidence supports that the SAARP can effectively target and damage a single ovary at a dose of 1 Gy.** Representative images of whole histological sections of ovaries from the (**a**) non-targeted (right) and (**b**) targeted (left) ovary of the same mouse. Examples of healthy antral follicles are highlighted with black asterisks and corpora lutea are highlighted with black diamonds. Corresponding higher magnification images in the boxed regions are shown in (**c-d**) to visualize primordial and primary follicles at the ovarian cortex. White asterisks in (**c**) highlight specific primordial follicles in the cortex that are absent in (**d**). The scale bar is 125 μm

**Fig. 6:**
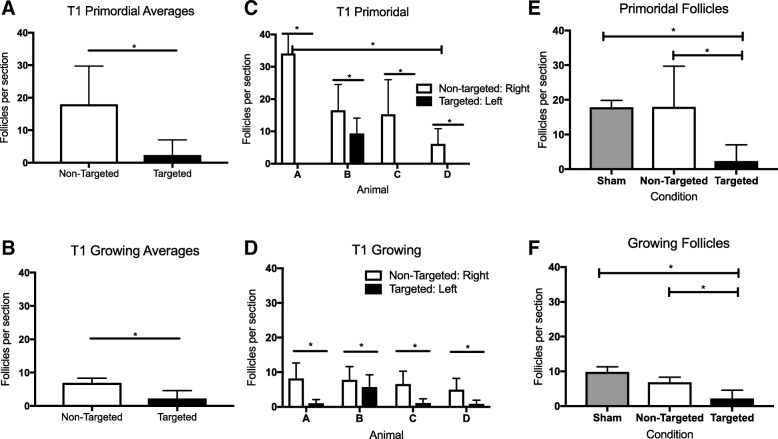
**Targeted radiation to a single ovary causes significant damage to the targeted ovary without having off target effects on the contralateral ovary.** The average number of (**a**) primordial follicles per section and (**b**) growing follicles per section were quantified in both the non-targeted and targeted ovaries. These data were delineated by individual animal (letter) (**c-d**). In (**e-f**), the same data that are shown in (**a-b**) were plotted alongside the average follicle counts for the Sham cohort shown in Fig. 3b-c. The data were analyzed either by unpaired t-tests or one-way ANOVAS. Statistical significance was defined as *p < 0.05

To examine potential off-target effects of radiation damage to the ovary, we used the SAARP to deliver precise radiation to one ovary while sparing the contralateral ovary for each mouse (Fig. 1a; T1 cohort). In this case, the non-targted ovary served as an internal control or a within-animal comparison, which is typically not possible, i.e., when only TBI is used. Based on gross assessment of ovarian histological sections, both non-targeted and targeted ovaries still had antral follicles and corpora lutea present in the tissue similar to what we observed in the TBI and T2 cohorts; providing evidence of follicle growth and estrous cyclicity (Fig. 5a and b). However, there was a visible difference in the primordial and primary follicles between the targeted and non-targeted ovaries. Whereas the non-targeted ovaries had clearly visible primordial and primary follicles at the cortex, the targeted ovaries did not (Fig. 5c and d). These observations were confirmed with follicle counting. When examining all follicle classes together, we observed that the targeted ovaries had significantly fewer total follicles relative to the non-targeted ovaries, which were similar to the total in Sham controls (1356.3 ± 2222.5 follicles, 6508.8 ± 2723.4 follicles, and 6689.2 ± 2289.6 follicles, respectively; Additional file 1). When focusing specifically on primordial follicles, we found that the targeted ovaries had significantly less primordial follicles per section compared to the non-targeted ovaries (2.3 ± 4.7 vs. 18.0 ± 11.7 primordial follicle per section, respectively, Fig. 6a). There were also fewer growing follicles in the targeted ovaries compared to the non-targeted ovaries (2.2 ± 2.4 growing follicles per section vs. 6.9 ± 1.5 growing follicles per section, respectively, Fig. 6b), and this was primarily attributable to changes in secondary follicles (Additional file 4). Interestingly, there were no changes in antral follicle counts in the different experimental cohorts, again suggesting that the largest follicles are not sensitive to radiation-induced damage (Additional file 4). To examine whether there was variability among mice, we examined follicle counts across individual animals (Fig. 6c-d, Additional file 4). While the number of primordial follicles in the non-targeted ovaries was similar in three of the four mice, mouse A had significantly more follicles than mouse D (34.1 ± 18.3 primordial follicles per section vs. 6.1 ± 4.7 follicles per section, respectively, Fig. 6c). There were no animal-specific differences in the number of growing follicles (Fig. 6d). Nevertheless, across animals, the targeted ovaries consistently exhibited a significant decrease in both primordial and growing follicle counts compared to their non-targeted counterparts (Fig. 6c and d). When further examining growing follicles according to specific follicle class, the targeted ovaries in each animal had significantly fewer primary and secondary follicles relative to the non-targeted ovary except for mouse B that had similar numbers of primary follicles in both ovaries (Additional file 4). Again across animals, there were no differences in antral follicles between the targeted and non-targeted ovaries (Additional file 4).

Although these results demonstrate that the SARRP is able to deliver precise radiation to a field as small as a single ovary, we wanted to assess whether there was any off-target damage to the non-targeted contralateral ovary. Therefore, we compared follicle counts in the T1 cohort (right ovary: non-targeted; and left ovary: targeted) to those in the Sham cohort (Fig. 6e-f). As expected, we found that when comparing the average primordial follicle counts in Sham controls to the targeted ovary in the T1 cohort, there was a marked decrease in primordial follicle counts: 17.8 ± 2.0 and 2.3 ± 4.7 primordial follicles per section, respectively (Fig. 6e). This decrease was also true for growing follicles where there were 9.9 ± 1.4 growing follicles per section in the Sham cohort but only 2.2 ± 2.4 growing follicles per section in the targeted ovaries from the T1 cohort (Fig. 6f). In contrast, there was no difference when comparing the average follicle counts in the Sham ovaries relative to the counts in non-targeted ovaries from the T1 cohort for either primordial or growing follicles, indicating negligible off target effects (Fig. 6e-f). Interestingly, when examining the growing follicles according to class, there did appear to be a decrease in primary follicles between the Sham and non-targeted cohorts, suggesting that primary follicles may be sensitive to off-target radiation effects (Additional file 4).

## Discussion

In this study, we used the SARRP to examine the effect of a single dose of ionizing radiation delivered to precise fields on ovarian damage in an adult whole animal mouse model. Although a limitation of our study is the small sample size in each experimental cohort, we nevertheless were able to draw important conclusions based on several robust findings. Overall, consistent with previous studies, we found that 1 Gy was sufficient to decrease follicle counts within the mouse ovary [28, 37, 39]. In fact, 1 Gy caused significant depletion of the ovarian reserve – or the number of primordial follicles – when delivered either as TBI or as targeted radiation to a field containing both ovaries (T2). This dose is much lower compared to humans where the effective sterilizing dose at which premature ovarian failure occurs immediately after treatment in 97.5% of patients is 16.5 Gy at 20 years and 14.3 Gy at 30 years [24]. Similarly, in nonhuman primates, a single dose of 15 Gy targeted radiation to the ovary resulted in a rapid decline in reproductive function [40]. Although we observed a significant reduction in the number of primordial follicles within the ovaries in the TBI and T2 cohorts, some follicles do remain. However, it is difficult to extrapolate whether this reduction would lead to partial or complete infertility in these mice because we do not know how the rate of follicle loss is affected when the ovarian reserve experiences such a significant depletion nor how residual follicles are recruited to contribute to fertility. In a recent study, cyclophosphamide-treated mice that showed a 90% reduction in follicle counts remarkably still maintained fertility [41]. However, previously we have shown that while follicles are still present following a single dose exposure of 1 Gy TBI at 2 weeks post-exposure, they are virtually all eliminated at 5 weeks post-exposure [37]. Thus, we anticipate that our treatment paradigm would likely affect fertility quite rapidly. Interestingly, we still observed the presence of antral follicles and corpora lutea in all the tissues we examined, and in fact, the number of antral follicles did not change across experimental cohorts. It takes approximately 18–24 days for a primordial follicle to reach the antral follicle stage in mouse [42]. Thus, any antral follicles or corpora lutea observed at the 2 week time point would be derived from a growing follicle that was present at the time of radiation and escaped damage to continue growth into a morphologically normal antral follicle.

Although the difference did not reach statistical significance, the TBI cohort tended to exhibit a more severe follicle loss phenotype compared to the cohort that received targeted radiation to both ovaries when considering both primordial and growing follicles. TBI could be more damaging to the ovary relative to targeted radiation due to compounding systemic effects. Radiation can induce systemic effects in which complex tissue responses are observed in non-irradiated regions due to signaling from irradiated cells. These systemic effects include inflammatory responses that may indirectly contribute to ovarian damage and follicle loss [15, 20, 22, 23, 43]. Ultimately, distinguishing the relative contribution of systemic damage compared to targeted damage in the ovary will require additional dose response studies with a larger sample size. Our findings parallel clinical data which demonstrated that female cancer survivors with spontaneous menstrual cycles who had been exposed to TBI and alkylating chemotherapy had a lower ovarian volume and follicle numbers compared to those who had received radiotherapy below the diaphragm with or without alkylating chemotherapy [5]. These findings suggest that TBI has a compounding effect on ovarian damage relative to targeted radiation. However, controlled human studies are nearly impossible due to variability in factors such as treatment regimen and radiation dose, further warranting animal studies and technologies such as the SARRP.

While systemic effects may account for the potential differences between the TBI and targeted radiation cohorts, it is also possible that individual animal variability contributes the extent of ovarian damage. For example, we observed that the larger number of remaining follicles in the cohort receiving targeted radiation to both ovaries compared to the TBI cohort was not a global phenomenon but rather due to the follicle numbers of two mice. This animal-specific response could be due to genetic variations in outbred CD-1 mice that confer differential radiosensitivity.

We also can not rule out the possibility that there could be technical inconsistencies with the targeting especially due to the challenging in vivo anatomy and small size of the mouse ovary. However, we do not think mis-targeting was a major limitation in our study. Geographic misalignment of the radiation field with respect to the ovary would be reflected in one of several possibilities. First, a portion of the ovary could have been outside the radiation field and, therefore, received less than the prescribed dose. Given the mm size of the ovary and the 1 cm field size, the probability is low that this would occur and result in a difference in dose (and therefore, damage and thus sequelae) within the ovary. However, if it did, this would be visible at the macroscopic level when individual ovaries (from either T1 or T2 protocols) were examined, and we did not observe evidence of a portion of the ovary being under-dosed. The second possibility is that ovary was completely outside the radiation field. If this were to happen in the T1 protocol, we would expect no reduction in follicles in the targeted ovary compared to the non-targeted ovary – since neither would have received radiation. This reduction was not observed. Rather, follicle number was substantially reduced in targeted ovaries relative to paired non-targeted ovaries. Conceivably, a misalignment of the 2 cm wide field could occur under the T2 protocol such that only one ovary received the full dose and the other received no dose (in the extreme situation). Misalignment would then result in a difference in follicle count between the two ovaries where there should not be such an effect. This difference was observed in one animal even though the follicle counts were obviously reduced from the shams, indicating a radiation effect. However, a difference between ovaries was also detected in one Sham animal. While inherent differences between ovaries within the same animal may make it more difficult to use a paired ovary as an internal control (T1 protocol), this variability was the exception rather than the rule and does not support the existence of dose inhomogeneity. Finally, the least likely scenario is that there was complete misalignment of the field during the T2 protocol such that both ovaries were completely excluded from the large radiation field. Although it is difficult to imagine how such a severe positioning error could have been made (and not noticed), there is one animal where the data would be consistent with this possibility. Because of this one potentially spurious result, we acknowledge the possibility of field misalignment as an unavoidable limitation of our experimental set-up.

Another key finding in our study was that significant follicle loss was often only observed in the targeted ovary when 1 Gy was delivered to a single ovary and not the contralateral ovary. This finding provides important proof-of-concept that the SAARP is a powerful method to target even the smallest structures within the mouse such as the ovary. In our experimental paradigm, we noted that the average primordial and growing follicle counts in the non-targeted ovaries were nearly identical to the Sham controls suggesting that the near complete loss of follicles in one ovary does not cause a dramatic change in the overall follicle profile of the remaining ovary despite individual animal variability in follicle numbers. In addition, the bystander effect appears to be negligible in our model. Although doses of 1 Gy can elicit bystander effects, this cell-signaling typically occurs through gap junction communication and paracrine signaling mechanisms which act over distances of a few millimeters. Thus, it is possible that paired mouse ovaries are too physically separated from each other to produce a biological effect. Another possibility is that 2 weeks post-radiation exposure is not sufficient to observe off target effects or compensatory effects in the non-targeted ovary that may take place over a longer time scale. In fact, we recently showed that a single dose exposure to 1 Gy TBI elicited significant germ cell damage without any overt stromal damage even up to 5 weeks post-exposure [44]. Thus, additional impacts of radiation damage that have been observed in human ovaries, such as stromal fibrosis and impaired vasculature, are late effects [4, 9, 14].

## Conclusion

Taken together our findings demonstrate the SARRP is an innovative tool to examine the impact and underlying mechanisms of targeted radiation damage as well as systemic effects. This system can be applied to modeling specific scenarios relevant to the field of fertility preservation. For example, it can be used to screen and determine the efficacy of fertoprotective agents against radiation damage in whole animal models [40, 45–47]. Furthermore, implementation of the SARRP will allow accurate modeling of clinical radiation therapies to better understand how they impact the ovary and reproductive function, thereby ultimately improving personalized medicine.

## Methods

### Animals

Female CD-1 mice (6 weeks old) from Envigo (Indianapolis, IN) were used in this study. Mice were housed in a controlled barrier facility at the University of Kansas Medical Center (KUMC) under constant temperature, humidity, and a 12 h light/dark cycle. Food and water were provided ad libitum. All animal experiments were approved by the Institutional Animal Care and Use Committee and were performed in accordance with the National Institutes of Health Guidelines.

### Irradiation procedure

Mice were randomized into experimental cohorts 1) those that were exposed to total body irradiation (TBI), 2) those that received radiation targeted to both ovaries (T2), and 3) those that received radiation targeted to one ovary (T1) (Fig. 1a). Sham mice served as controls. Between 3 and 5 mice were used in each experimental cohort. The Xstrahl Small Animal Radiation Research Platform (SARRP, Xstrahl Inc., Suwanee, GA) was used to deliver a conformal dose of 1 Gy at a dose rate of 0.037 Gy/sec with an exposure time of 27 s to a designated field (Fig. 1b-c). Mice were anesthetized using a subcutaneous injection of buprenorphine (0.05–0.1 mg/kg) and then placed on the irradiator platform (Fig. 1b). The mice in the TBI group were irradiated in a field containing the whole mouse with the collimator removed. Mice in the T2 cohort were irradiated in a 20 mm × 10 mm collimated field containing both ovaries, whereas mice in the T1 cohort had only the left ovary exposed to radiation in a 10 mm × 10 mm collimated field (Fig 1c). The spine and the knee served as landmarks for defining the exposure field. Sham mice were handled the same way as those that were irradiated (i.e. handled, anesthetized, placed in the microirradiator) but were not exposed to radiation. Mice in all cohorts were monitored post-exposure to ensure that there were no visible acute effects of radiation and were weighed and euthanized 2 weeks post-procedure.

### Ovarian tissue fixation and histological processing

Ovaries were harvested from all mice, and the bursa was removed by careful dissection under a light microscope. The right and left ovaries for each mouse were processed separately. Ovaries were fixed in Modified Davidson’s fixative which is a prepared combination of 14% ethyl alcohol, 37.5% formalin, 37–39% glacial acetic acid, in 1 L of deionized water (Electron Microscopy Sciences, Hatfield, PA). Ovaries were rocked gently in fixative for 6 h at room temperature and then transferred to 4 °C overnight (~ 15 h). Samples were washed in 70% ethanol and processed and dehydrated using an automated tissue processor (Leica Biosystems, Buffalo Grove, IL). Ovaries were placed in molds and embedded in paraffin wax (Leica EG1160, Leica Biosystems, Buffalo Grove, IL). Paraffin embedded ovarian blocks were serial sectioned at a thickness of 5 μm using a Leica RM2335 microtome (Leica Biosystems, Buffalo Grove, IL). Every fifth section was placed on a separate slide designated for hematoxylin and eosin (H&E) staining to facilitate follicle counting. Stained sections were counted as a proxy for ovarian size to determine any differences between experimental groups (Fig. 1e). Slides were H&E stained using the AutostainerXL (Leica Biosystems, Buffalo Grove, IL). Whole slides were digitally imaged at the University of Washington Histology and Imaging Core using the Hamamatsu-HT imaging system (Hamamatsu Photonics, Hamamatsu City, Japan). NanoZoomer Digital Pathology Software (Hamamatsu Photonics) was used to analyze the images for follicle counting.

### Follicle classification and counting

We used established morphological criteria to classify and count healthy follicles [38]. Atretic follicles with pyknotic granulosa cells or abnormal oocytes were excluded. In brief, a primordial follicle was defined as an oocyte surrounded by a complete or incomplete layer of squamous granulosa cells; a primary follicle was defined as an oocyte surrounded by a single complete layer of cuboidal granulosa cells; a secondary follicle was defined as an oocyte surrounded by two or more layers of granulosa cells; finally, antral follicles were defined as an oocyte surrounded by multiple layers of granulosa cells and containing a clear fluid filled cavity. Secondary and antral follicles were only counted if the oocyte nucleus was visible to avoid double counting. Follicles were grouped into two classes: primordial and growing. Growing follicles were defined as any follicle that was primary, secondary, or antral. Follicles were counted on every 5th section and either reported as total counts per experimental cohort or the number of follicles per total number of sections counted for each respective ovary.

### Statistics

Follicle counts were analyzed based on cohort, animal, and ovary. Data represented in all graphs represent the mean ± standard deviation. Comparisons of two independent groups were done using unpaired t-test, and comparisons of more than two independent groups were done using a one-way ANOVA test followed by a post-hoc test. Statistical significance was defined as *p* < 0.05. All analyses were completed using GraphPad PRISM software (GraphPad Software Inc., La Jolla, CA).

## Additional files

Additional file 1: Total follicle counts. The total number of follicles (primordial, primary, secondary, and antral) were quantified, and the average total number per each experimental cohort (Sham, TBI, T2, and T1) are plotted. A one-way ANOVA was performed between cohorts, and statistical significance was defined as *p < 0.05. (PDF 480 kb)

Additional file 2: The effect of TBI and targeted radiation on growing follicles analyzed by specific class. The average number of (A) primary follicles per section, (B) secondary follicles per section, and (C) antral follicles per section were quantified in ovaries from each cohort (Sham, TBI, and T2). A one-way ANOVA was performed between cohorts, and statistical significance was defined as *p < 0.05. (PDF 480 kb)

Additional file 3: Individual animal variation in growing follicle numbers in response to radiation damage. The growing follicle data shown in Fig. 4 was further broken down into follicle classes, including (A-C) primary follicles, (D-F) secondary follicles, and (G-I) antral follicles. Data for the Sham cohort are shown in A, D, G, for the TBI cohort in B, E, H, and for the T2 cohort in C, F, and I. Data for individual animals are denoted by letters and for the right and left ovaries by black and white bars, respectively. Unpaired t-tests were performed to compare follicle counts between the right and left ovaries with statistical significance defined as *p < 0.05. (PDF 480 kb)

Additional file 4: Stage-specific effects on growing follicles of targeted radiation to a single ovary relative to its non-targeted contralateral counterpart. The average number of (A) primary follicles per section, (B) secondary follicles per section, and (C) antral follicles per section were quantified in both the non-targeted and targeted ovaries and compared to the Sham cohort. These data were also delineated by individual animal (letter) (D-F). The data were analyzed either by unpaired t-tests or one-way ANOVAS. Statistical significance was defined as *p < 0.05. (PDF 480 kb)

## Abbreviations

H&E: Hematoxylin and eosin
HPG axis: Hypothalamus-pituitary-gonad axis
RIBE: Radiation-induced bystander effect
SARRP: Small Animal Radiation Research Platform
T1: Radiation targeted to one ovary
T2: Radiation targeted to both ovaries
TBI: Total body irradiation
