# Supplementary figures and images for: Use of a Small Animal Radiation Research Platform (SARRP) facilitates analysis of systemic versus targeted radiation effects in the mouse ovary
Source: J Ovarian Res. 2018 Aug 30;11:72. doi: 10.1186/s13048-018-0442-8 (PMC6116356; doi:10.1186/s13048-018-0442-8)

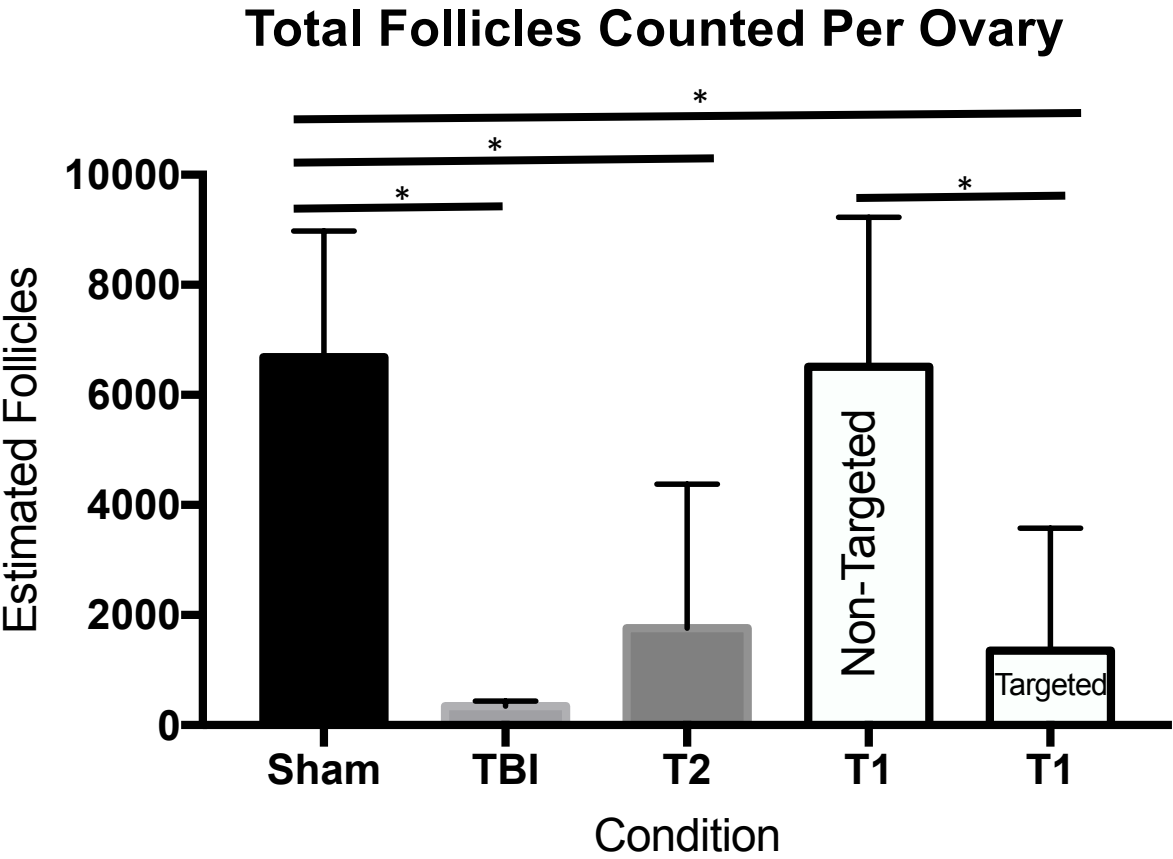

Supplement: Supplementary file 1 — Total follicle counts. The total number of follicles (primordial, primary, secondary, and antral) were quantified, and the average total number per each experimental cohort (Sham, TBI, T2, and T1) are plotted. A one-way ANOVA was performed between cohorts, and statistical significance was defined as *p < 0.05. (PDF 480 kb) [file 13048_2018_442_MOESM1_ESM.pdf]

Additional File 2

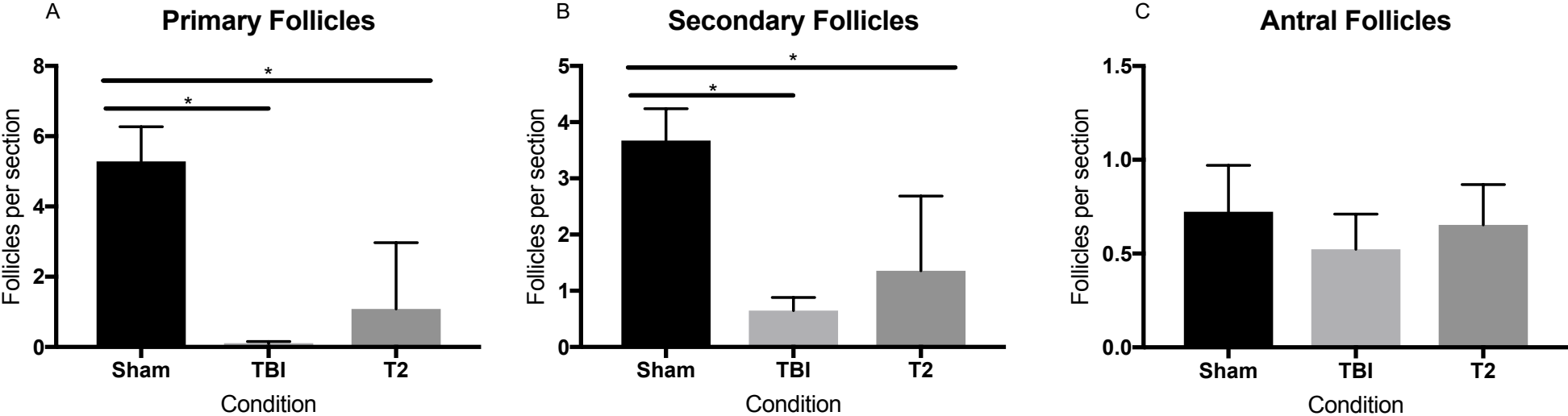

Supplement: Supplementary file 2 — The effect of TBI and targeted radiation on growing follicles analyzed by specific class. The average number of (A) primary follicles per section, (B) secondary follicles per section, and (C) antral follicles per section were quantified in ovaries from each cohort (Sham, TBI, and T2). A one-way ANOVA was performed between cohorts, and statistical significance was defined as *p < 0.05. (PDF 480 kb) [file 13048_2018_442_MOESM2_ESM.pdf]

# Additional File 3

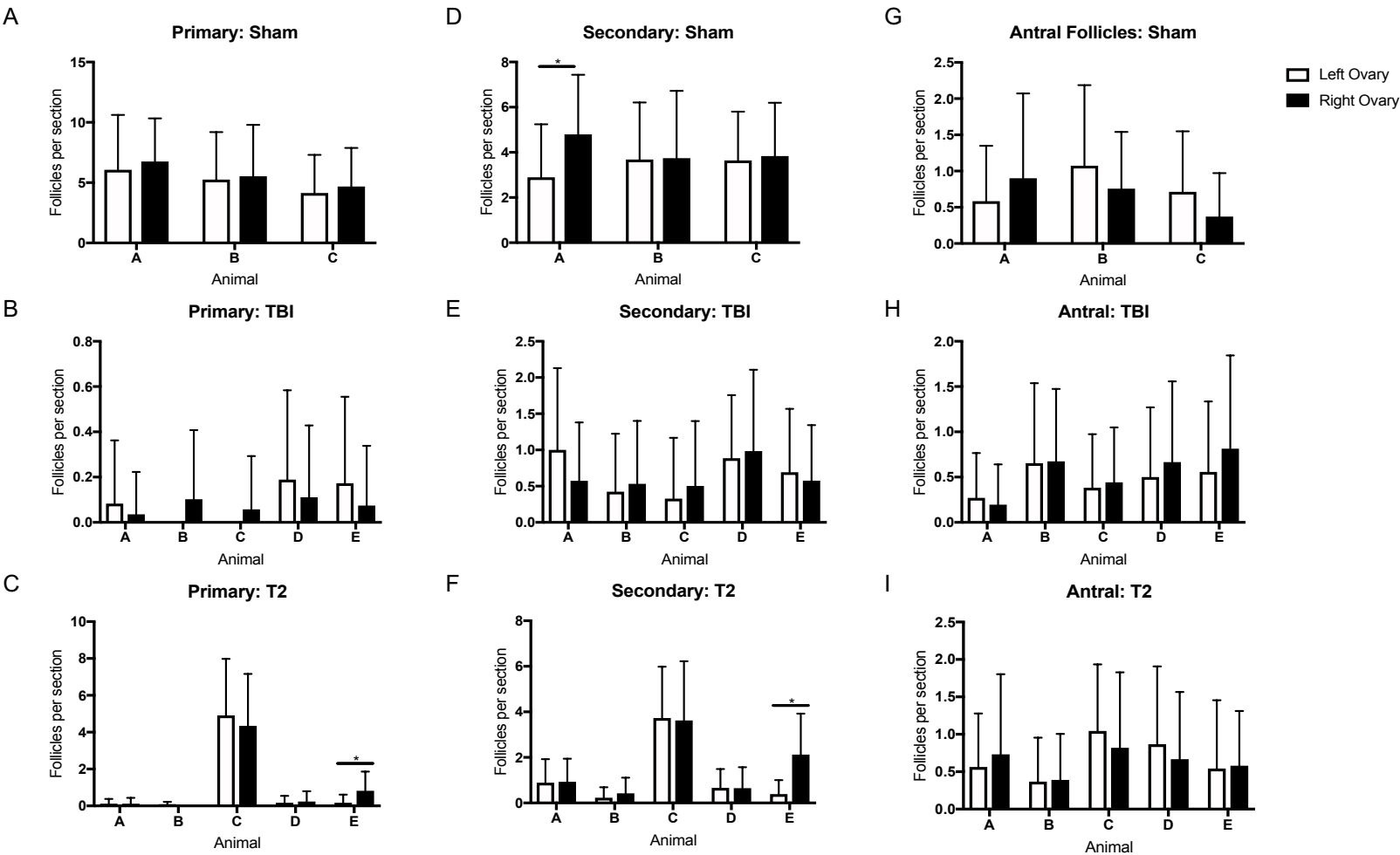

Supplement: Supplementary file 3 — Individual animal variation in growing follicle numbers in response to radiation damage. The growing follicle data shown in Fig. 4 was further broken down into follicle classes, including (A-C) primary follicles, (D-F) secondary follicles, and (G-I) antral follicles. Data for the Sham cohort are shown in A, D, G, for the TBI cohort in B, E, H, and for the T2 cohort in C, F, and I. Data for individual animals are denoted by letters and for the right and left ovaries by black and white bars, respectively. Unpaired t-tests were performed to compare follicle counts between the right and left ovaries with statistical significance defined as *p < 0.05. (PDF 480 kb) [file 13048_2018_442_MOESM3_ESM.pdf]

Additional File 4

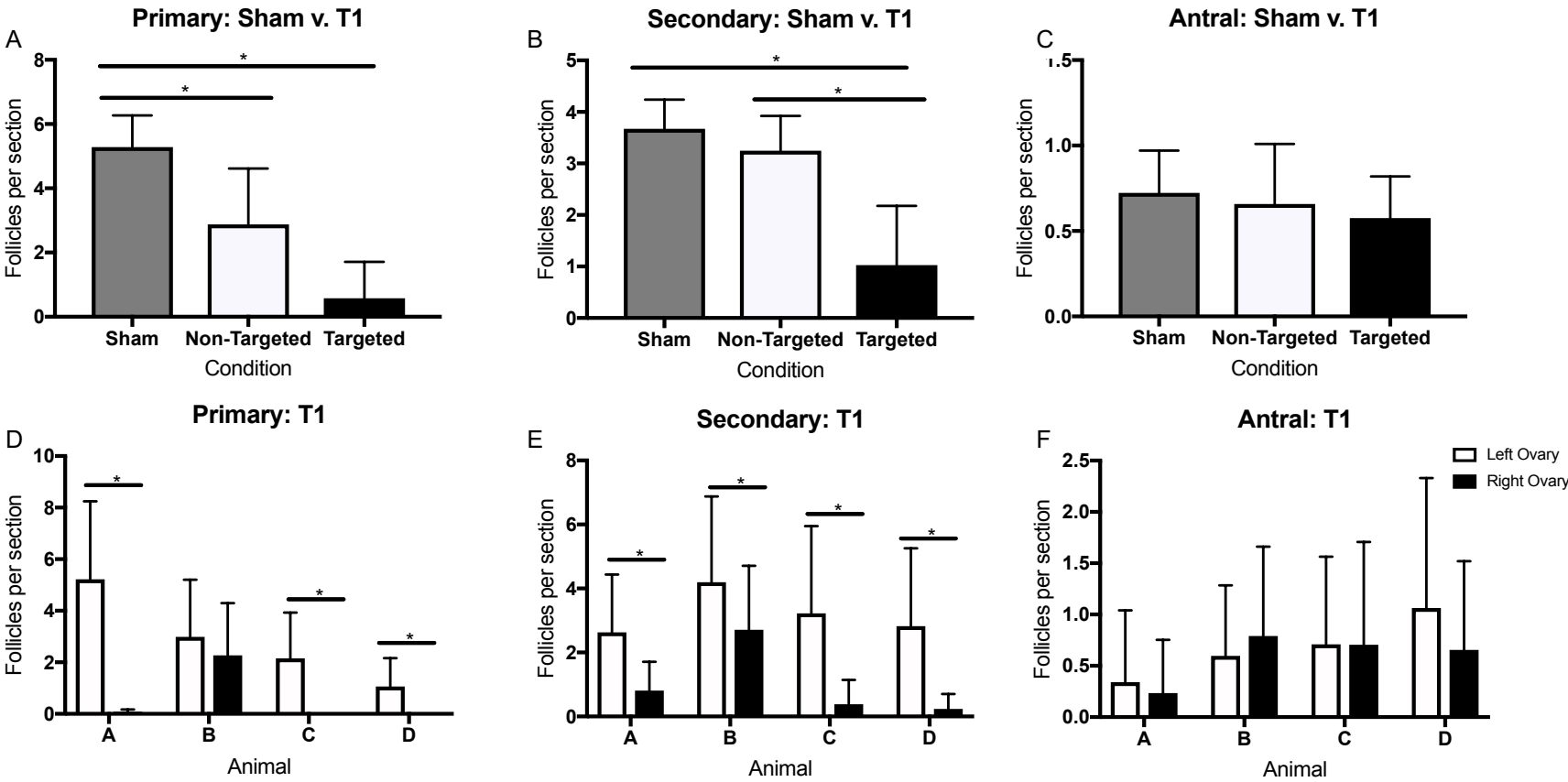

Supplement: Supplementary file 4 — Stage-specific effects on growing follicles of targeted radiation to a single ovary relative to its non-targeted contralateral counterpart. The average number of (A) primary follicles per section, (B) secondary follicles per section, and (C) antral follicles per section were quantified in both the non-targeted and targeted ovaries and compared to the Sham cohort. These data were also delineated by individual animal (letter) (D-F). The data were analyzed either by unpaired t-tests or one-way ANOVAS. Statistical significance was defined as *p < 0.05. (PDF 480 kb) [file 13048_2018_442_MOESM4_ESM.pdf]
